# **Impact of nano-cellulose fiber addition on physico-mechanical properties of room temperature vulcanized maxillofacial siliconematerial**

**DOI:** 10.1016/j.jtumed.2023.07.002

**Published:** 2023-07-16

**Authors:** Ashraf Abdulrazzaq Ali, Ihab Nabeel Safi

**Affiliations:** Department of Prosthodontics, College of Dentistry, University of Baghdad, Baghdad, Iraq

**Keywords:** الاستطالة, الألياف النانوية, صلابة, خشونة, التمزق وقوة, الشد, Elongation, Nanofiber, Hardness, Roughness, Tear, Tensile

## Abstract

**Objectives:**

Maxillofacial silicone is used to restore abnormalities due to congenital or acquired causes. However, the quality of silicone is far from ideal. This study was aimed at assessing the influence of the addition of cellulose nanofibers (CNFs; several nanometers in diameter and 2–5 μm long) on the physical and mechanical characteristics of maxillofacial silicone elastomers.

**Methods:**

Two CNF weight percentages (0.5% and 1%) were tested, and 180 specimens were divided into one control and two experimental groups. Each group was subdivided into six subgroups. In each subgroup, ten specimens subjected to each of the following tests: tearing strength, Shore-A hardness, tensile strength, elongation percentage, surface roughness, and color stability. The samples were additionally analyzed with Fourier transform infrared spectroscopy (FTIR) and field emission scanning electronic microscopy (FESEM).

**Results:**

The 0.5% CNF group, compared with the control group, exhibited highly significantly greater tearing strength, elongation percentage, hardness Shore-A, and surface roughness, and substantially greater tensile strength. However, color stability did not significantly differ between groups.

The 1% CNF group showed significantly greater Shore-A hardness, tear strength, color stability, and surface roughness, and insignificantly lower tensile strength and percentage elongating values, than the control group. FESEM imaging revealed good CNF dispersion. The FTIR spectra indicated that CNFs interacted with silicone through surface functional hydroxyl groups.

**Conclusion:**

Addition of 0.5 wt. % CNF to silicone elastomers increased the material's mechanical tensile strength, tear strength, elongation percentage, and hardness as long as it stayed within the acceptable range for clinical use. Surface roughness increased in direct proportion to the amount of nanofibers added. Moreover, addition of 0.5 wt. % CNF to silicone polymers had insignificant effects on color stability.

## الملخص

**أهداف البحث:**

يستخدم سيليكون الوجه والفكين لاستعادة التشوهات الناتجة عن أسباب خلقية أو مكتسبة. ومع ذلك، فإن جودة السيليكون بعيدة كل البعد عن المثالية. تهدف هذه الدراسة إلى تقييم تأثير ألياف السليلوز النانوية (ذات قطر نانومتري والطول 2-5 ميكرومتر) بالإضافة إلى الخصائص الفيزيائية والميكانيكية لسيليكون اللدائن المرنة للوجه والفكين.

**طريقة البحث:**

تم استخدام نسبتين من الوزن (0,5% و 1%). تم إنشاء 180عينة وتصنيفها إلى مجموعة ضابطة ومجموعتين تجريبيتين. تم تقسيم كل مجموعة إلى ست مجموعات فرعية. لكل مجموعة فرعية، تم استخدام 10 عينات لكل اختبار (قوة التمزق، قياس صلابة المواد، قوة الشد، نسبة الاستطالة، خشونة السطح، ثبات اللون). تم فحص العينات بواسطة المجهر الالكتروني الماسح والتحليل الطيفي للأشعه تحت الحمراء لتحويل "فورييه” والانبعاثات الميدانية.

**النتائج:**

أظهرت المجموعات 0,5% زيادة معنوية عالية في مقاومة التمزق ونسبة الاستطالة وصلابة المواد وخشونة السطح وارتفاع كبير في اختبار مقاومة الشد مقارنة بمجموعة التحكم. ومع ذلك، لم يختلف ثبات اللون بشكل كبير بين المجموعتين. بينما أظهرت المجموعة التجريبية 1%زيادة معنوية في صلابة المواد،مقاومة التمزق، ثبات اللون، خشونة السطح وانخفاض غير معنوي في مقاومة الشد وقيم الاستطالة المئوية بالمقارنة مع مجموعة التحكم العادية. أظهرت صور المجهر الالكتروني الماسح توزيع جيد لألياف السليلوز النانوية مع زيادة ظهور التكتلات عند التراكيز العالية للألياف النانوية وكما أظهر التحليل الطيفي للأشعه تحت الحمراء الى تفاعل ألياف السليلوز النانوية مع السيلكون من خلال مجموعات الهدروكسيل الوظيفيه السطحية.

**الاستنتاجات:**

أدت إضافة 0,5% بالوزن من ألياف السليلوز النانوية إلى سيليكون الوجه والفكين إلى زيادة قوة الشد الميكانيكية للمادة، وقوة التمزق، ونسبة الاستطالة والصلابة طالما بقيت ضمن النطاق المقبول في الاستخدام السريري. زادت خشونة السطح بالتناسب المباشر مع كمية الألياف النانوية المضافة. علاوة على ذلك، فإن إضافة 0,5% بالوزن من ألياف السليلوز النانوية إلى بوليمر السيليكون له تأثير ضئيل على ثبات اللون.

## Introduction

Congenital flaws, trauma, or tumor surgery can all result in facial deformities. Surgical reconstruction may not be possible, depending on the defect size and location. However, a prosthetic that recapitulates the natural characteristics of the missing tissues from acquired, developmental, and congenital head and neck disorders may be able to improve the appearance and correct functional defects.^1^

Silicone elastomers are the primary materials used in maxillofacial prosthesis applications. Their physical characteristics and favorable flexibility make them suitable for applications in which the flexibility of silicone accommodates movement of soft tissue; these materials also have favorable biocompatibility, longevity, chemical inertness, easy manipulation and comfort.^2^

Versiltal silicone elastomer room temperature vulcanized VST-50 (RTV) maxillofacial silicone has many desirable properties. The main drawbacks associated with silicone are low tensile strength and tear strength; insufficient elasticity; and deterioration of mechanical, physical, and color properties during aging.^3^

Given the psychological and social consequences on patients, increasing the mechanical properties and color stability of the silicone elastomers used in maxillofacial prostheses is critical. Several techniques have been used to enhance silicone elastomers' features. One such technique involves the inclusion of nanofibers or nanofillers to increase the material's tear and tensile strength, and improve its physical and mechanical characteristics, thus making it more useful in therapeutic settings.^4^^,^^5^

Natural plant fibers have recently received substantial attention, owing to their exceptional qualities, such as being non-biohazardous, affordable, biocompatible, and derived from renewable resources. Cellulose is a substance comprising a linear chain of hundreds to tens of thousands of 1–4-linked D-glucose units, with a chemical formula of (C_6_H_10_O_5_)_n_. As a novel environmentally friendly material, cellulose nanofibers (CNFs) are expected to be extensively used in a variety of industries, such as healthcare and the reinforcement of polymers. The extremely high Young's modulus, and tensile and tear strengths of the fibers, owing to their small size, enable new alternative reinforcing methods. Wound dressings and tissue engineering are notable areas in the biomedical sector in which nanocellulose is an extremely suitable biomaterial.^6^^,^^7^

In 2022, Leite et al.^6^ found that the Vickers hardness can be increased by the simple addition of CNCs, a renewable material. Adding CNCs to this denture reline resin may increase the material's abrasion resistance, thus supporting long-term use.

The aim of this study was to add organic nanofibers to silicone elastomers at 0.5 and 1 wt. %, to enhance the mechanical and physical properties of the maxillofacial silicone polymer. The null hypothesis was that incorporating CNF into maxillofacial silicone elastomer would not have adverse effects on the properties of the silicone, whereas the alternative hypothesis was that incorporating CNF with silicone elastomer would enhance the properties of the silicone.

## Materials and Methods

### Sample preparation

Maxillofacial silicone replacement material was vulcanized at room temperature (RTV; VST-50, Factor II Inc., USA) and mixed with CNF powder from Nanografi Nano Technology Co., Germany (100 nm fiber size, 2–5 μm length, 1.50 g/cm^3^, white color, and 329 °C decomposition temperature).

Three groups of samples were analyzed: (1) Control: neat silicone samples without CNFs; (2) Group I: silicone samples with 0.5 wt. % CNFs; (3) Group II: samples of silicone with 1 wt. % CNFs.

Each set (specific group) contained 61 samples, which were divided into ten samples for each of the following tests: mechanical tearing strength, tensile strength, Shore-A hardness, elongation percentage, surface texture roughness, and color stability. One representative sample from each group was subjected to field emission scanning electron microscopy (FESEM).

The samples in the control group were prepared by combining the silicone base components. A flowable paste material comprised part A, and tetraethyl orthosilicate crosslinked platinum catalyst material in liquid form comprised part B. The weight proportion was 10:1 (as recommended by the manufacturer). In brief, 200 g silicon base and 20 g of catalyst were combined for 5 min with a mixer under vacuum suction (Multivac 3, Degussa, Germany). For samples in groups I and II, the silicone base was mixed with the CNF powder to form the modified silicone base, then mixed with a vacuum mixer for 12 min. The vacuum was turned off for the first 5 min to avoid suction of CNF powder and was then turned on for the next 7 min at a speed of 360 rpm and a vacuum value of −10 bar to remove air bubbles and disperse CNF into the silicone polymer.^8^^,^^9^

**Figure 1:**
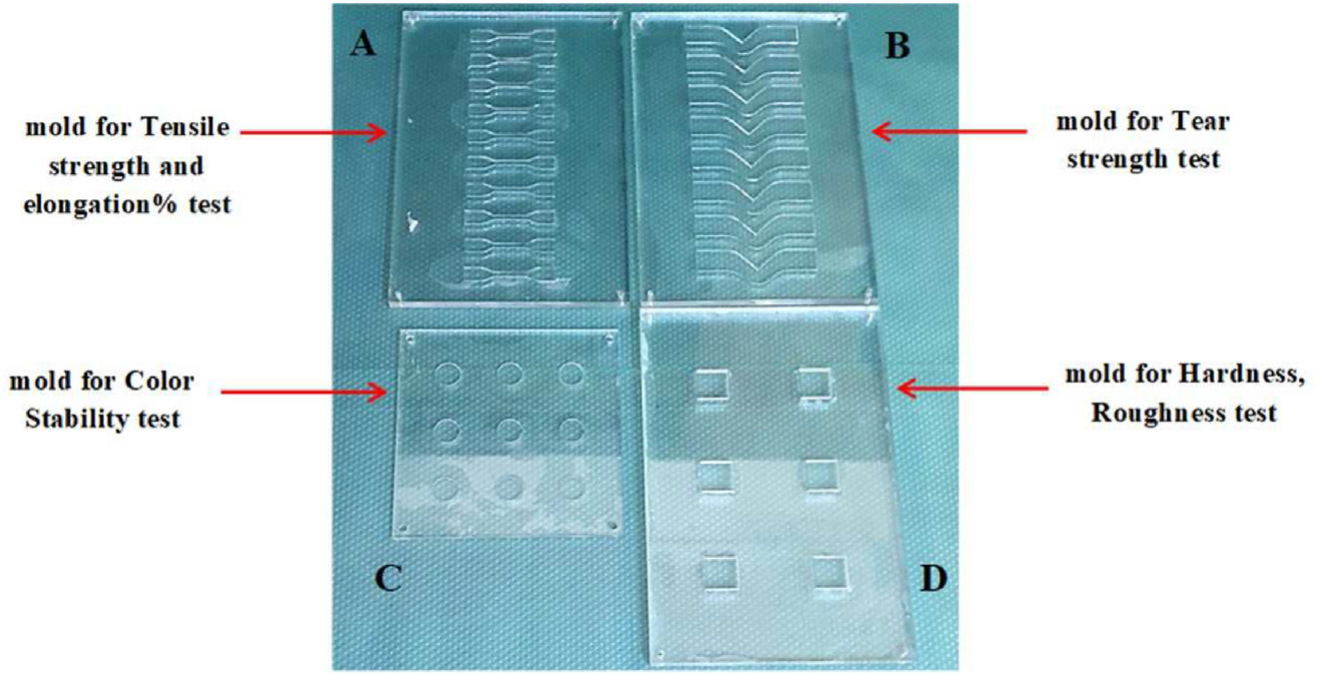
Plastic mold: A) 2 mm deep mold for tensile strength testing, B) 2 mm deep mold for tear strength testing, C) 2 mm deep mold for color stability testing, D) 6 mm deep mold for hardness and roughness testing.

**Figure 2:**
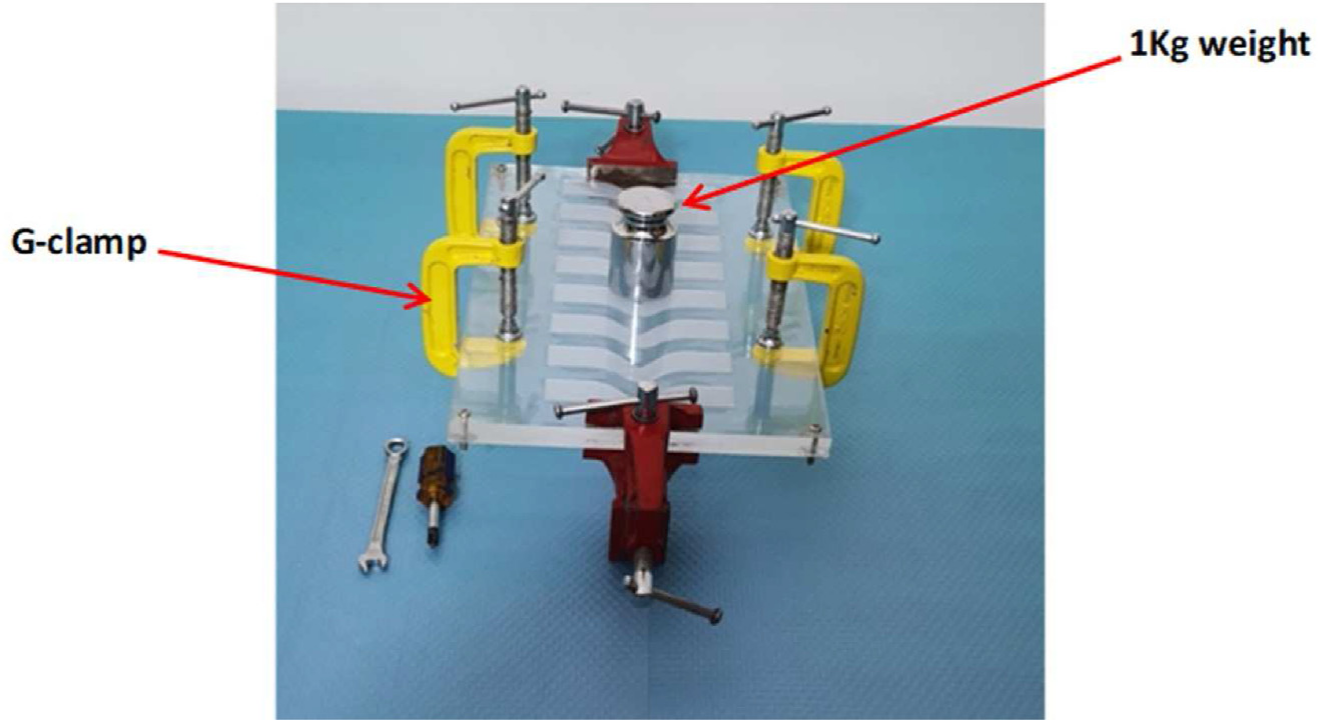
A mold fixed with screws and nuts at the corners with six G-clamps, 1 kg weight.

Specimens were formed in the template holes of plastic molds (Figure 1). A center plastic rim was attached to the lower plate, and the upper and lower plastic plates composed the mold. The mixture was poured into the mold and allowed to set for 24 h after the mold was fixed with screws, washers, and nuts at the corners. The material was squeezed with six G-clamp holders at 1 kg weight (Figure 2). The samples were formed into 2 mm-thick specimens for testing of mechanical tearing and tensile strength, color stability, and experimental elongation percentage, and into 6 mm-thick specimens for evaluation of hardness and surface texture roughness (Figure 1). After vulcanization, the specimens were withdrawn from the mold and placed in light-proof containers before testing. The specimens were kept in the storage box under as controlled an atmosphere as possible for a minimum of 16 h; the temperature was kept between 20 °C and 25 °C, the humidity was kept below 60%, and special care was taken to avoid stacking the specimens on top of one another. The specimens were also shielded from light exposure throughout the vulcanization and testing processes.^4^^,^^10^

### Tensile strength testing

**Figure 3:**
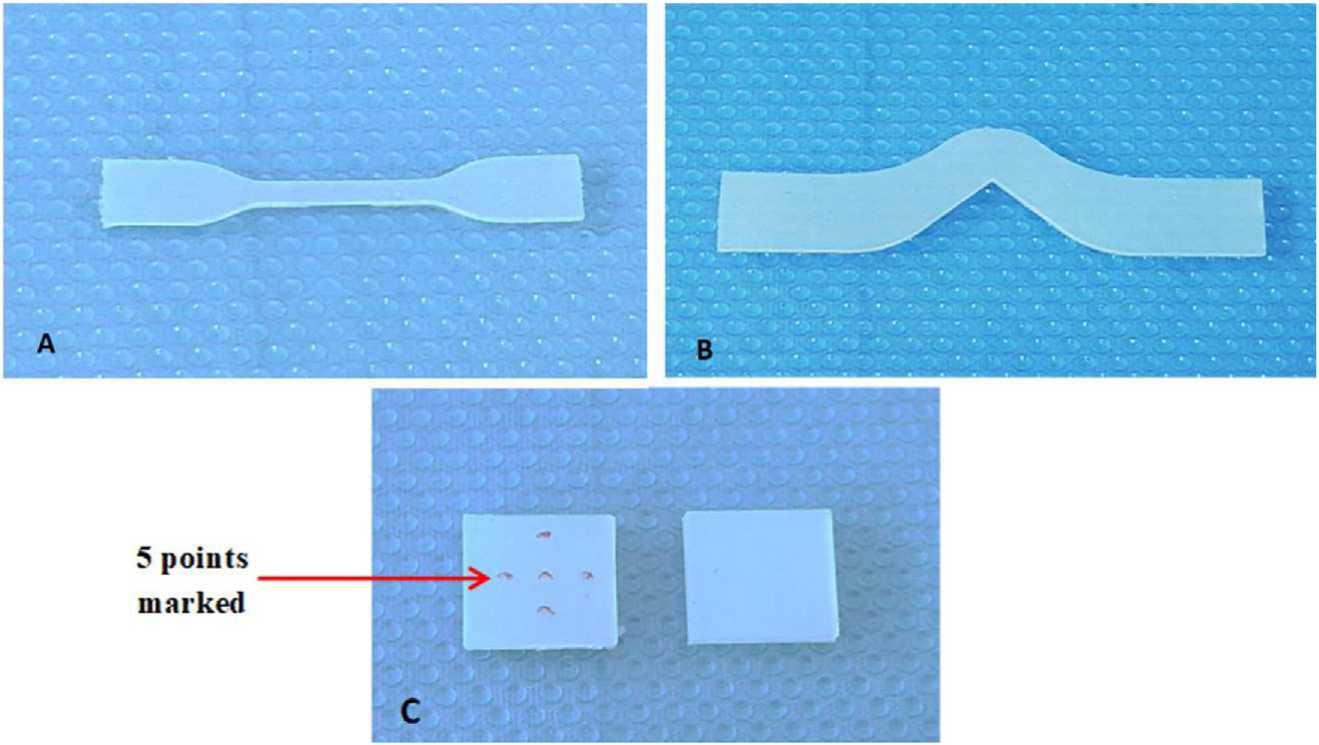
Design of silicone specimens: A) pattern for the tensile strength testing, B) pattern for mechanical tearing strength testing, C) pattern for Shore-A hardness testing.

Dumbbell-shaped type 2 samples (75 mm length, 2 mm thickness) were used for tensile strength testing (Figure 3A) in accordance with ISO 37:2017.^11^ Extensometers were used to clamp the samples to the universal testing device (GESTER-Techno Co. Ltd. China). The maximum force was measured at 500 mm/min until breakage. The samples' narrow section width (w) and thickness (t) were measured with digital calipers (in millimeters). The strongest possible force at break (F) was expressed in Newtons. The tensile strength was calculated with the following equation and expressed in MPa:

Ts = F/wt

### Tear strength testing

ISO 34-1:2015 was followed for the tear testing.^12^ With an unnicked angle-shaped die, sample dimensions of 102 mm length and 2 mm thickness were created from a plastic mold (Figure 3B). The samples were fastened to a universal testing apparatus (GESTER-Techno Co. Ltd., China) and stretched at 500 mm per minute until failure. The strongest possible force (F) at cutoff was recorded in Newtons, and digital electronic calipers were used to measure the sample thickness (t) in the area of the right angle, in millimeters. Tear strength (T, in N/mm) was estimated as follows: T = F/t.

### Shore-A hardness testing

A digital durometer with Shore-A scale (HS-A digital scale; Ezitown, China) was used for hardness measurement according to ISO 48-4:2018.^13^ The samples were 25 mm × 25 mm square. On one surface of each sample, five point marker indentations (with a diameter of 1.25 mm) were created with the blunt indenter of the durometer, and the average of hardness was calculated. The gap distance between the borders of each depression and the sample was 6 mm (Figure 3C).

### Elongation percentage testing

According to the length at cutoff (Lb) and the starting length (Lo) of the sample's narrowest section, the elongation percentage was computed in accordance with ISO 37:2017.11. To calculate the elongation at cutoff, the below equation was used:

percentage elongation = Lb − Lo/Lo × 100

where Lo is the starting length (mm), and Lb is the length after breaking (mm).

### Surface roughness

The sample design was a square shaped surface with dimensions of 25 mm × 25 mm.^13^ The surface texture roughness was measured with a digital profilometer (Ra, TR 220, Beijing High Technology Ltd., China). The stylus analyzer in the profilometer was shifted by an 11 mm distance on the surface of the silicone sample; this parameter was the average of a set of single measurements of surface peaks and valleys. Three measurements of the surface roughness Ra were collected, and the average values were calculated and are reported as the roughness values.

### Color stability testing

Theisc samples prepared for color change studies were 20 mm in diameter and 2 mm in thickness.^14^ Digital images were acquired from control and experimental specimens to distinguish the contrast from the transparent specimens of the silicone samples. A digital imaging approach with a single-lens reflex camera (Canon, Japan) and a 105 mm camera macro lens (Sigma, Japan) was used.^14^

**Figure 4:**
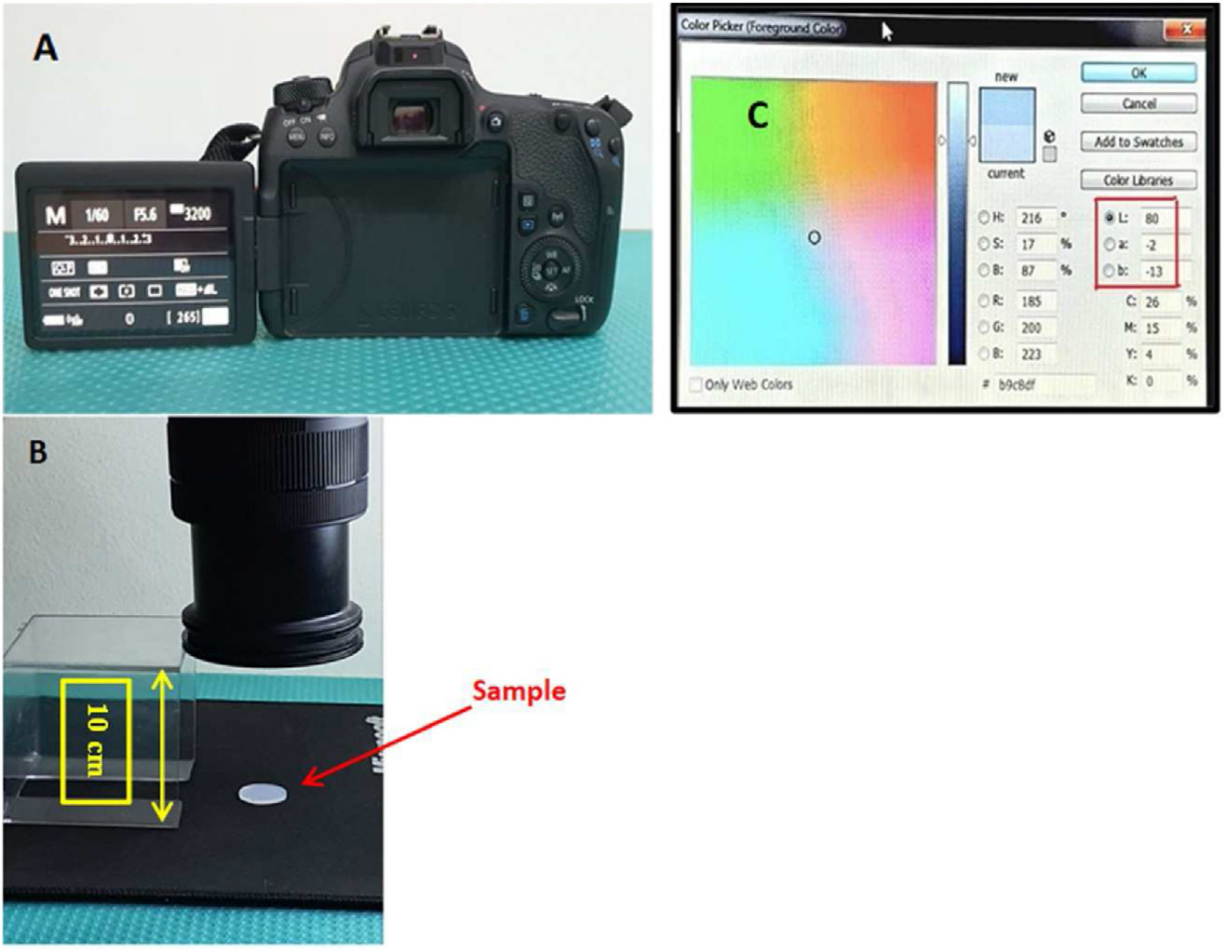
A) Digital camera setting. B) The camera was fixed perpendicularly at a 10 cm distance from the specimen. C) Reading in the color picker palette tab for (L, a & b) parameters.

The digital camera was mounted perpendicularly on a stand clamp holder and set to manual mode to control the shutter speed, ISO, and f-stop settings of 1/60 and 5.6. During photography, these measurements remained constant (Figure 4 A, B).

The digital photographs were downloaded to a laptop and saved as TIFF files. Adobe Photoshop CS6, Ver. 13.0.1, was used to evaluate the images (Adobe Systems, USA). Red, green, and blue values were extracted and converted into (L-a-b) or (v-h-c) values through mathematical modeling.^14^

The RGB Lab system was used in the color study, and the Commission Internationale de l'Eclairage (CIE) was used to assess color change (ΔE). Lightness value (L), hue (a), and chroma (b) were measured to assess the color stability of each sample after the addition of CNF to VST-50 silicone.^15^ The silicone sample was attached and removed from the surveyor's table, which had been placed in a stable location; the sample was positioned parallel to the camera lens between time points to ensure optimal and repeatable positioning for each measurement.^16^

A measurement template with a 60-pixel square area was made in the center of the sample for standardizing calculations. The color information was collected directly from the L, a, and b parameters’ color picker palette tab.^14^ (Figure 4, C).

Color coordinates (L, a, and b) of each sample were measured in three groups as follows:

Ι. At baseline: measurement was performed before any treatment (control group) of silicone (L0, a0, and b0).
II. After addition of 0.5% CNF (experimental group): color coordinates were measured as L1, a1, and b1.
III After addition of 1% CNF (experimental group): color coordinates were measured as L2, a2, and b2.

### Fourier transform infrared spectroscopy testing

Fourier transform infrared spectroscopy testing (FTIR) was used to evaluate whether silicone polymer (VST-50) and CNF interact chemically. Three samples were examined: the modified groups with the inclusion of CNFs; the silicone-only control group; and the CNF powder alone group. To determine the effects on specimens, FTIR analysis was conducted. The CNF powder quantity was just sufficient to cover the device lens, and the silicone specimens were created as thin pieces 10 × 10 × 0.5 mm in length, breadth, and thickness, respectively.^17^

### Field emission scanning electron microscopy testing

The 2 mm specimens were cut with a sharp knife into 10 mm square cross-sectional parts. A single sample from each of the control group, experimental group, and CNF powder group was sputter coated with gold. The samples were analyzed with a field emission scanning electron microscope (INSPECT F50, Netherlands) to determine the distribution of CNFs throughout the silicone matrix.

### Statistical analysis

Prism 9 (GraphPad Software, USA) and SPSS were used for data analysis (Statistical Package for Social Science, version 21). The results are displayed as bar charts with mean values and standard deviations for descriptive analysis. Multiple comparisons were performed with one-way ANOVA and the post-hoc Tukey's HSD test. Non-significant, significant, and highly significant differences were defined by P-values >0.05, <0.05, and <0.01, respectively.

## Results

Each experimental groups (group I with 0.5% CNFs and group II with 1% CNFs) was compared with the control group without CNFs.

Silicone rubber samples reinforced by CNF addition were analyzed. FTIR spectra were collected to assess changes in the chemical structure and functional groups.

The characteristic OH stretching was visible in the IR spectra of CNF powder at 3330 cm^–1^, and C–OH bending and C=O stretching were responsible for peaks at 1700–1500 cm^−1^.

**Figure 5:**
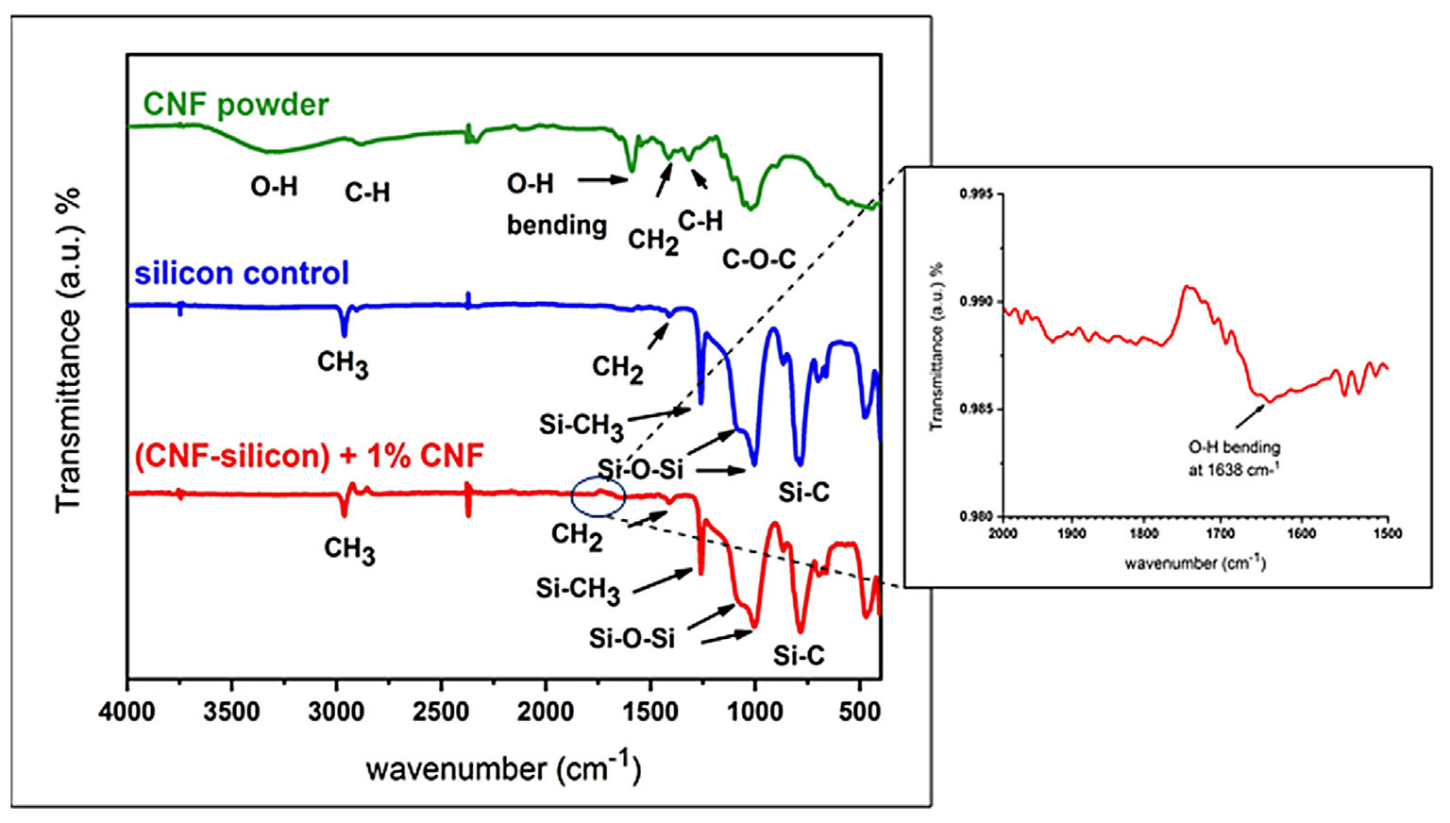
IR spectra of CNF, silicon control, and CNF/silicon composite.

IR spectra for silicone reinforced by CNF were recorded. The peak associated with –OH stretching at 3321 cm^−1^ disappeared, and a peak assigned to –OH bending appeared at 1638 cm^−1^ in the silicone rubber samples reinforced by CNFs (FTIR spectra; Figure 5).

**Figure 6:**
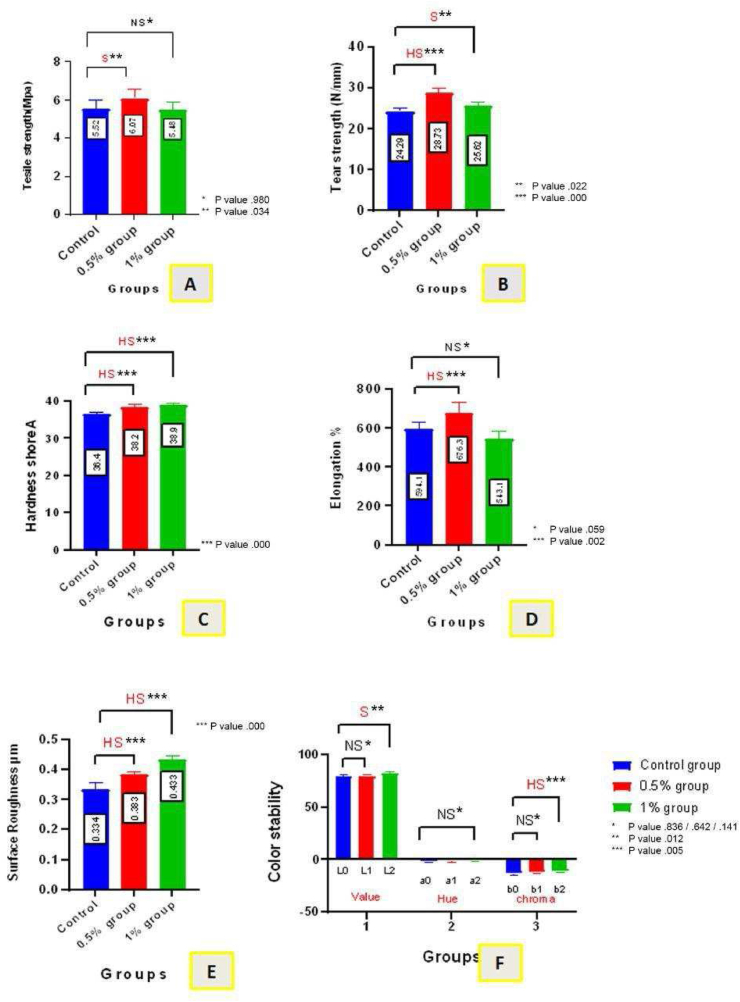
Bar chart for: A) tensile strength testing, B) tear strength testing, C) hardness Shore-A testing, D) elongation percentage testing, E) surface roughness testing, and F) color stability testing.

The mean values in experimental group I were higher than those in the control group, whereas the mean values in experimental group II were lower than those in the control group. Group II had the lowest mean tensile strength (5.48 MPa), whereas group I had the highest mean value (6.072 MPa); the mean value of the control group was (5.52 MPa). According to one-way ANOVA, the differences were significant. According to Tukey's HSD test, a significant difference (p < 0.05) between group I and the control group was found, whereas no difference was observed between group II and the control group. The tensile strength data are shown in Figure 6, A.

The control group's mean tear strength (24.29 N/mm) was lower than those of the two experimental groups (group I = 28.73 N/mm; group II = 25.62 N/mm) as shown in Figure 6, B. One-way ANOVA revealed a highly significant difference (p < 0.01) between the test groups. A highly significant difference (p < 0.01) between group I and the control group, as well as a significant difference between group II and the control group, were found with Tukey's HSD test.

The Shore-A hardness mean values in the experimental groups were higher than those in the control groups (Figure 6, C). The control group's mean hardness (36.4) had the lowest value, whereas group II had the highest mean hardness (38.9), and group I had an average hardness of 38.2. A substantial difference was observed among groups, according to the ANOVA results. Tukey's HSD test showed a highly significant difference between each experimental group and the control group.

Group I's mean value (676.333%) was highest with respect to that in the control group (594.166%). The lowest elongation percentage (543.166%) was observed in group II. A significant difference between test groups was determined with one-way ANOVA. Tukey's HSD test revealed a highly significant difference between the means of group I and the control group. A non-significant difference (p > 0.05) was found between the means of group II and the control group. Figure 6, D shows the data on elongation at break.

The control group had a surface texture roughness value of 0.334 μm, whereas groups I and II had values of 0.383 and 0.433 μm, respectively. One-way ANOVA indicated a substantial difference between the test groups. Tukey's HSD test revealed a statistically significant difference between the control group and group I or II. The surface roughness outcomes are shown in Figure 6, E.

For color data analyses of color stability, means and standard deviations were calculated for dependent variables ΔL∗, Δa∗, and Δb∗ The value (L) of color stability testing and the mean values are presented in (Figure 6, F). The mean value in group II was highest (81.8), and was followed by those in group I (79.6) and the control group (79.1).

The outcomes of the one-way ANOVA revealed significant differences between the test groups. Tukey's HSD test results showed a non-significant difference between the control group (L0) and the experimental group I (L1), but a significant difference between the control group (L0) and the experimental group II (L2).

The descriptive statistics of hue (a) in color stability analyses are shown in (Figure 6, F). Group II had the greatest mean value (−0.8) and was followed by group I (−1), whereas the control group displayed the lowest mean value (−1.4). One-way ANOVA revealed statistically non-significant differences between the test groups.

The descriptive statistics for color stability chroma (b) were also determined. The control group had the lowest mean value (−13.2), followed by group I (−12), whereas group II had the highest mean value (−11.1). One-way ANOVA revealed a substantial difference between test groups. According to Tukey's HSD test, no significant difference was observed between the control group (b0) and the experimental group I (b1); however, a substantial difference was found between the control group (b0) and the experimental group II (b2; Figure 6, F).

ΔE∗ = [(ΔL∗)^2^ + (Δa∗)^2^ + (Δb∗) 2]^1/2^. ^14^

ΔE∗(0.5%) = [(L0 − L1)^2^ + (a0 − a1)^2^ + (b0 − b1)^2^]^1/2^

 = [(79.1 − 79.6)^2^+ (−1.4 − (−1))^2^ + (−13.2 − (−12))^2]1/2^

 = [(−0.5)^2^ + (−0.4)^2^ + (−1.2)^2^]^1/2^

 = [0.25 + 0.16 + 1.44]

 = [1.85]^1/2^

ΔE∗(0.5%) = 1.36

ΔE∗(1%) = [(L0 − L2)^2^ + (a0 − a2)^2^ + (b0 − b2)^2^ ]^1/2^

 = [(79.1 − 81.8)^2^ + (−1.4 − (−0.8))^2^ + (−13.2 − (−11.1))^2^ ]^1/2^

 = [(−2.7)^2^ + (−0.6)^2^ + (−2.1)^2^]^1/2^

 = [7.29 + 0.36 + 4.41]^1/2^

 = [12.06]^1/2^

ΔE∗(1%) = 3.47

**Figure 7:**
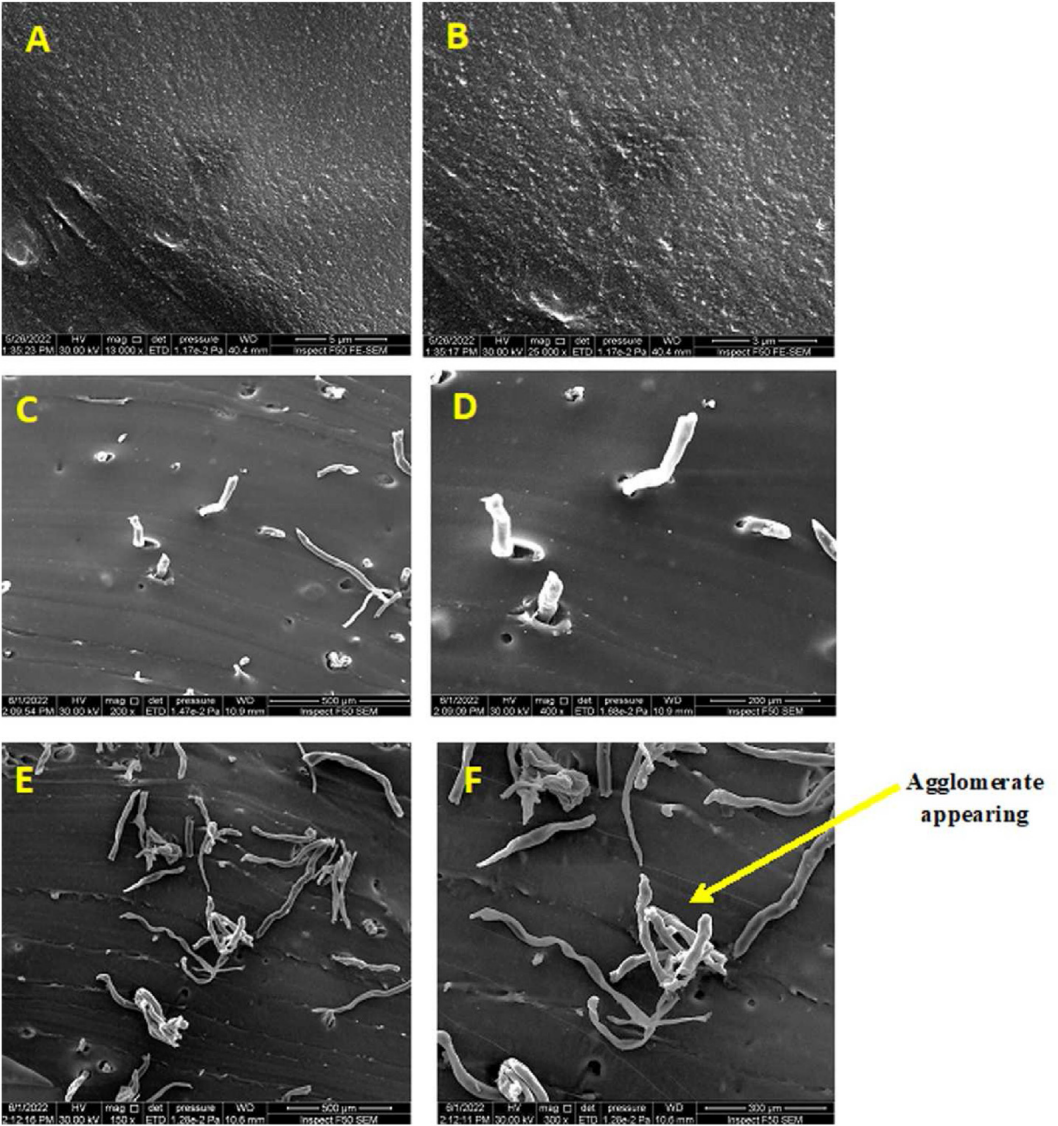
FESEM images (cross section) demonstrating uniform CNF distribution with an increase in the amount of large CNF agglomerates with increasing nanofiber loading. (A, B) set of control group; (C, D) 0.5% set of group I; (E, F) 1% set of group II.

FESEM pictures revealed that the silicone matrix reinforced by CNFs in all experimental groups had an excellent distribution of nanofibers due to the shear mixing vacuum, and group II had more agglomerates than the other groups (Figure 7).

## Discussion

Silicone rubber (VST-50) is one of the most common and cost-effective RTV silicone elastomers.^18^ No commercially available maxillofacial material can support the demands of daily prosthesis wear.^19^, ^20^, ^21^ Reinforcement with a specific amount of fiber is required to achieve a silicone polymeric material's required mechanical strength.^22^, ^23^, ^24^

Nanocellulose fibers have been used because of their good physical and mechanical characteristics (high hardness, rigidity, and thermal stability).^6^^,^^25^ Nanocellulose fibers are also abundant; can be obtained from a variety of plant sources; and offer a sustainable, environmentally beneficial, and inexpensive reinforcing option.^26^

The FTIR spectrum of CNF powder showed OH stretching fluctuation at 3330 cm^−1^ and OH bending vibration at 1647 cm^−1^. After addition of CNF to VST-50 silicone (RTV), the peak of OH stretching vibration faded, and its intensity decreased. The OH bending fluctuation at 1647 cm^−1^ in the CNF spectrum showed new peak formation after CNF was added to the silicone polymer, as a result of interactions of the functional –OH groups of CNF with one another, thus resulting in physical binding and interaction with the pair electron of each O present in silicon (Figure 5). Moreover, the existence of many active functional –OH groups leads to Van de Waals forces and hydrogen bond formation, thus resulting in physical interactions (molecular interactions) that enhance the binding strength, thereby increasing the shear strength and adhesive forces.^27^

CNFs form multifunctional cross-links that increase tensile strength through the formation of strong hydrogen bonds between the surface hydroxyl group and the silicone chains. The polymer becomes more stiff and durable because of these multifunctional cross-links, which also increase the overall cross-linking density and tensile strength.^28^ The mean tensile strength increased when 0.5% CNF was incorporated, because the acceleration of elastomer crystallization through short-fiber inclusion significantly affects tensile strength, and this strain-induced crystallization enhances tensile properties.^29^

The 1% CNF group showed diminished tensile strength, because of the high CNF loading. Fiber–fiber interaction aggregates bound by weak Van de Waals forces resulted in an agglomerated morphology, thus causing the chains to break more quickly under tensile force.^30^

The increase in tear strength with 0.5% CNF loading was due to the chemical and physical interactions of CNFs with the polymer chains. Through inhibition of both the movement of polymer segments against the nanofiber surface and the movement of other polymer chains against one another, these trapped networks increased the total network density and significantly increased the stiffness of the polymer.^31^, ^32^, ^33^

The diminished tear strength in the 1% CNF group was due to the nanofibers beginning to agglomerate. As the polymer is exposed to external forces, the agglomerates break and weaken the matrix, thereby causing the tear to spread. These agglomerates function as stress focusing sites inside the polymer matrix.^30^

The hardness test indicates a material's softness and flexibility, which should mimic that of the surrounding facial tissues. The acceptable Shore-A hardness values for maxillofacial prosthesis material should be in the range of 10–45 IU, depending on the missing facial part.^34^ The modulus of elasticity of the CNFs is higher than that of the silicone material, thus increasing the stiffness of the silicone matrix and the indentation resistance similarly to reinforced filler. The nanofibers were evenly spread throughout the polymer (FESEM in Figure 7) and gradually formed networks inside the polymer, thus decreasing the inter-aggregate space, and increasing the stiffness and hardness of the material.^5^ All experimental groups showed increased Shore-A hardness with CNF addition. This elevation of Shore-A hardness was directly proportional to the CNF content, because of the creation of a fiber–fiber mesh within a polymer matrix and the evenly distributed stiff and rigid CNF.^35^^,^^36^

The mean tensile strength and elongation percentage increased when CNF was incorporated because of acceleration of the crystallization behavior of short fibers in elastomers. This strain-induced crystallization improved the tensile and elongation properties. The presence of fibers in higher concentrations resulted in narrower distances between the polymer chains, thus limiting the versatility of the silicone elastomer.^29^

The decrease in the tensile strength and mean elongation percentage after addition of 1% CNFs restricted the chain-level mobility of the polymers, because of the generation of multifunctional cross-links, thus decreasing the stretching ability of the material.^37^

CNF has short whiskers sticking out from its surface. During sample preparation, the CNF fibers were presumed to have been distributed at random. The increase in mean surface roughness after addition of CNFs may be attributable to these various orientations across the surface as well as the projecting whiskers that were dispersed across the silicone surface. The increase in surface roughness of the silicone might have been caused by the beginning of agglomeration of fibers on the sample surfaces as the concentration of added fibers increased.^38^

One reason for prosthesis replacement is color change, which occurs as a result of aging, the use of chemicals or disinfectants, or both.^39^

After capturing pictures on a light-sensing medium, digital cameras produce images with red, green, and blue RGB values for every pixel.^40^

For Δb∗(chroma) color change, negative Δb∗ values denoted increased bluish color after addition of CNF. Addition of 0.5% CNF did not result in a significant difference in bluish color, whereas addition of 1% CNF resulted in a significantly more bluish chroma than that in the control group.

As the load on the fibers increases, a denser network forms within the matrix of the polymer. The fibers tend to fill in any gaps or empty spaces in the polymer. The amount of light transmitted may be constrained as a result of the interaction of light with the polymer, thus potentially resulting in some light being partially absorbed and some being partially reflected.^9^

The decreased light transmission is attributable to the CNF's scattering effect. Light scattering occurs because the nanofiber has a different index of refraction from that of the silicone elastomer. The scattering effect causes the material to show less translucency and to have a lighter appearance. All reinforced groups had a slightly lighter color value than the unreinforced control group, with greater chroma of the color. The increase in color intensity (chroma) is due to light absorption or scattering effects of nanofibers.^14^

The FESEM images demonstrated that the nanofibers had been successfully incorporated into the silicone material through high-shear mixing and a vacuum suction mixer. They also demonstrated a well-dispersed 0.5 wt. % concentration of CNF without agglomeration. When the weight-based concentration of nanofibers reached 1%, CNFs were well dispersed with some agglomeration. Further studies may investigate the influences of adding CNFs on the mechanical properties and color stability of maxillofacial silicone VST-50 (RTV) after different periods of accelerated artificial aging, and the influences on bacterial and candidal adherence after the aging process.

## Conclusion

Incorporating different weight percentages of CNFs (0.5–1 wt. %) into substitution silicone material VST-50 (RTV) significantly enhanced its mechanical characteristics (tearing and tensile strength, elongation percentage at cutoff). Optimum improvement was obtained at a concentration of 0.5 wt. % CNF, with no effect on the translucency of the silicone material. CNFs increased the hardness and surface roughness of the silicone in a manner directly proportional to CNF concentration.

## Source of funding

No specific grant for this research was provided by funding organizations in the public, commercial, or nonprofit sectors.

## Conflict of interest

All authors disclose that they have no conflicts of interest.

## Ethical approval

No ethical approval was applicable, because this was an experimental laboratory study.

## Author contributions

INS designed the study. AAA conducted the research, and collected and organized the data. AAA provided research materials. INS analyzed and interpreted the data. AAA wrote the initial and final drafts of the article. All authors have critically reviewed and approved the final draft, and are responsible for the content and similarity index of the manuscript.

## Source of data availability

The authors confirm that the data supporting the findings of this study are available within the article and its supplementary material. Raw data that support the findings of this study are available from the corresponding author upon reasonable request.
