# Supplementary material for: Impact of nano-cellulose fiber addition on physico-mechanical properties of room temperature vulcanized maxillofacial siliconematerial
Source: J Taibah Univ Med Sci. 2023 Jul 16;18(6):1616–26. doi: 10.1016/j.jtumed.2023.07.002 (PMC10497998; doi:10.1016/j.jtumed.2023.07.002)
Supplement: Multimedia component 1 [file mmc1.pdf]

Appendix 1: Data representing values of tensile strength test in MPa:

NO.	Control	CNF 0.5%	CNF 1%
1	5.77	6.11	5.48
2	6.22	6.2	6.22
3	5.55	6.33	5.11
4	5.11	5.88	6
5	5.77	5.55	5.33
6	5.55	6.22	5.55
7	5.88	5.55	5
8	4.66	6	5.66
9	4.88	7.22	5.22
10	5.88	5.66	5.31
**AVG**	5.527	6.072	5.488

Appendix 2: Data representing tear strength test readings (N/mm).

NO.	Control	CNF 0.5%	CNF 1%
1	23.8	30.2	24.4
2	25.3	27.6	25.7
3	24	27.2	24.8
4	24.7	30.1	27.2
5	24.2	27.1	25.5
6	25.5	30.6	26.8
7	24.3	27.8	25.9
8	23.2	28.5	24.2
9	23.5	29.3	25.1
10	24.4	28.9	26.6
**AVG**	24.29	28.73	25.62

Appendix 3: Data representing values of shore A hardness test (IU):

NO.	Control	CNF 0.5%	CNF 1%
1	36.8	38.8	38.2
2	36.2	36.8	38.8
3	36	38.4	39.2
4	35.2	39.2	39.1
5	36.4	37.2	38.8
6	37.8	40	39.6
7	35.6	37.6	39.3
8	36.4	37.4	39.2
9	37.2	38.4	38.2
10	36.4	38.8	38.6
**AVG**	36.4	38.26	38.9

Appendix 4: Data representing values of elongation percentage test:

NO.	Control	CNF 0.5%	CNF 1%
1	565.833	693.333	551.666
2	551.666	665	523.333
3	636.666	608.333	495
4	665	622.5	466.666
5	580	636.666	565.833
6	622.5	750	537.5
7	594.166	721.666	551.666
8	537.5	608.333	608.333
9	580	679.166	551.666
10	608.333	778.333	580
AVG	594.1664	676.333	543.1663

Appendix 5: Data representing values of surface roughness test readings in (µm):

NO.	Control	CNF 0.5%	CNF 1%
1	0.305	0.371	0.429
2	0.315	0.369	0.465
3	0.318	0.374	0.414
4	0.318	0.398	0.447
5	0.335	0.394	0.429
6	0.338	0.387	0.441
7	0.360	0.390	0.415
8	0.370	0.383	0.434
9	0.330	0.384	0.428
10	0.359	0.380	0.431
**AVG**	**0.334**	**0.383**	**0.433**

Appendix 6: Data representing the value (L) of color stability test:

NO.	Control	CNF 0.5%	CNF 1%
1	75	79	85
2	81	79	78
3	80	80	81
4	79	80	80
5	80	81	83
6	78	80	81
7	77	79	85
8	82	80	79
9	80	77	82
10	79	81	84
**AVG**	**79.1**	**79.6**	**81.8**

Appendix 7: Data representing the hue (a) of color stability test:

NO.	Control	CNF 0.5%	CNF 1%
1	-3	-2	-2
2	-2	-3	-2
3	1	-2	-2
4	-2	-2	-1
5	-2	-1	-1
6	-3	-1	1
7	-1	1	0
8	-1	2	0
9	1	-3	-1
10	-2	1	0
**AVG**	**-1.4**	**-1**	**-0.8**

Appendix 8: Data representing the chroma (b) of color stability test:

NO.	Control	CNF 0.5%	CNF 1%
1	-12	-13	-13
2	-13	-14	-11
3	-13	-12	-12
4	-14	-11	-12
5	-13	-11	-10
6	-15	-12	-12
7	-16	-13	-10
8	-10	-13	-11
9	-12	-11	-10
10	-14	-10	-10
**AVG**	**-13.2**	**-12**	**-11.1**

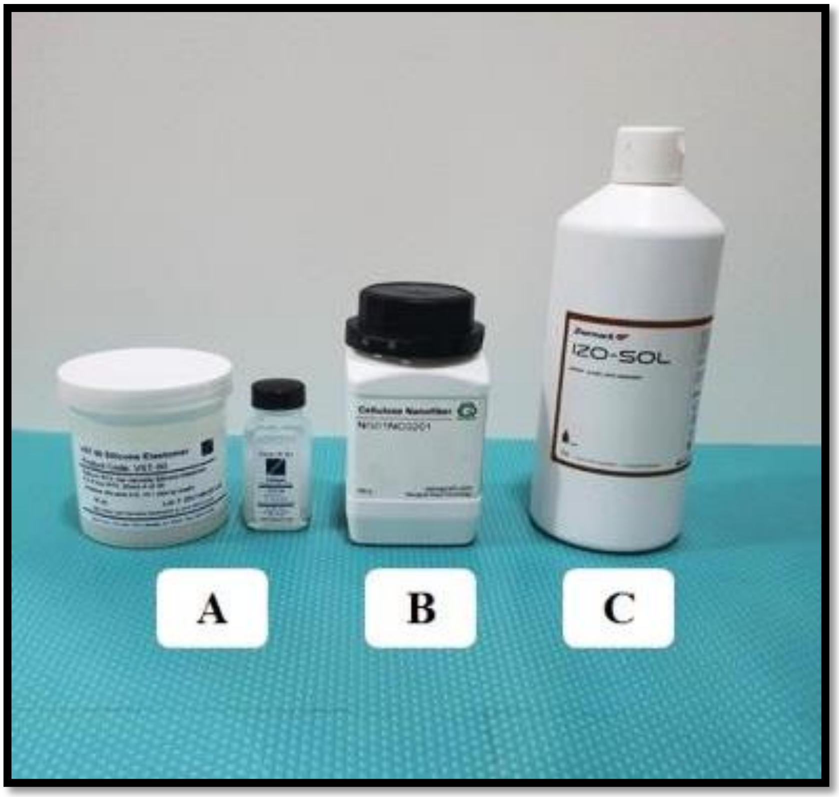

Some of the material used in study: A) VST50 silicone base and catalyst, B) Cellulose nanofiber, C) Separating medium.

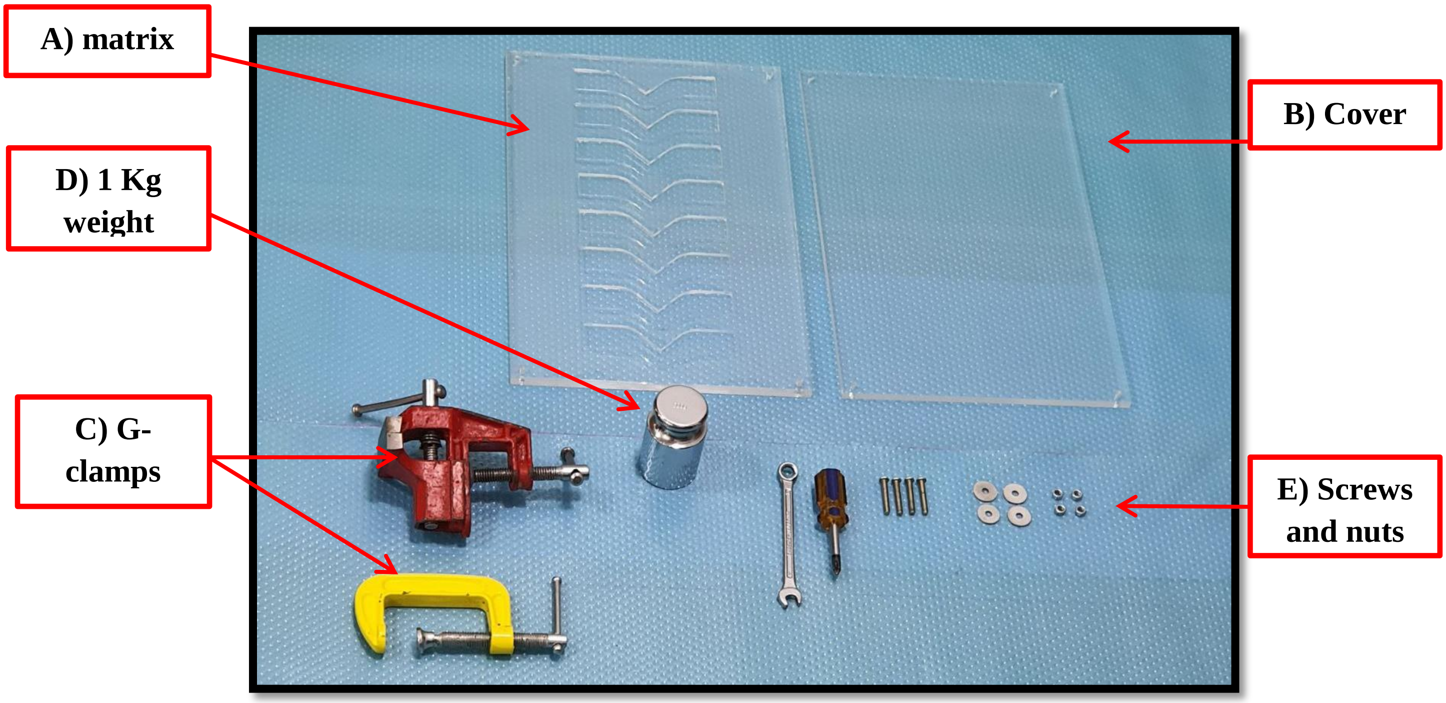

Parts of the mold: A) Matrix, B) Cover, C) clamps, D)1 kg weight, E) Screws, Washers, Nuts, spanner.

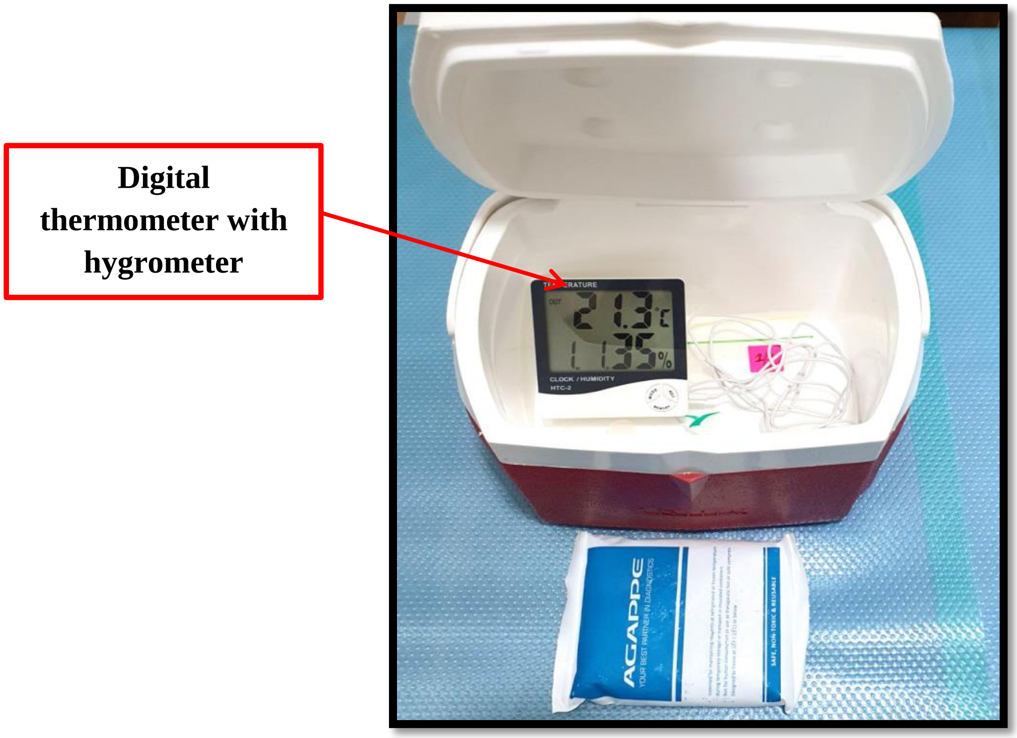

Vaccine storage box, a digital thermometer with

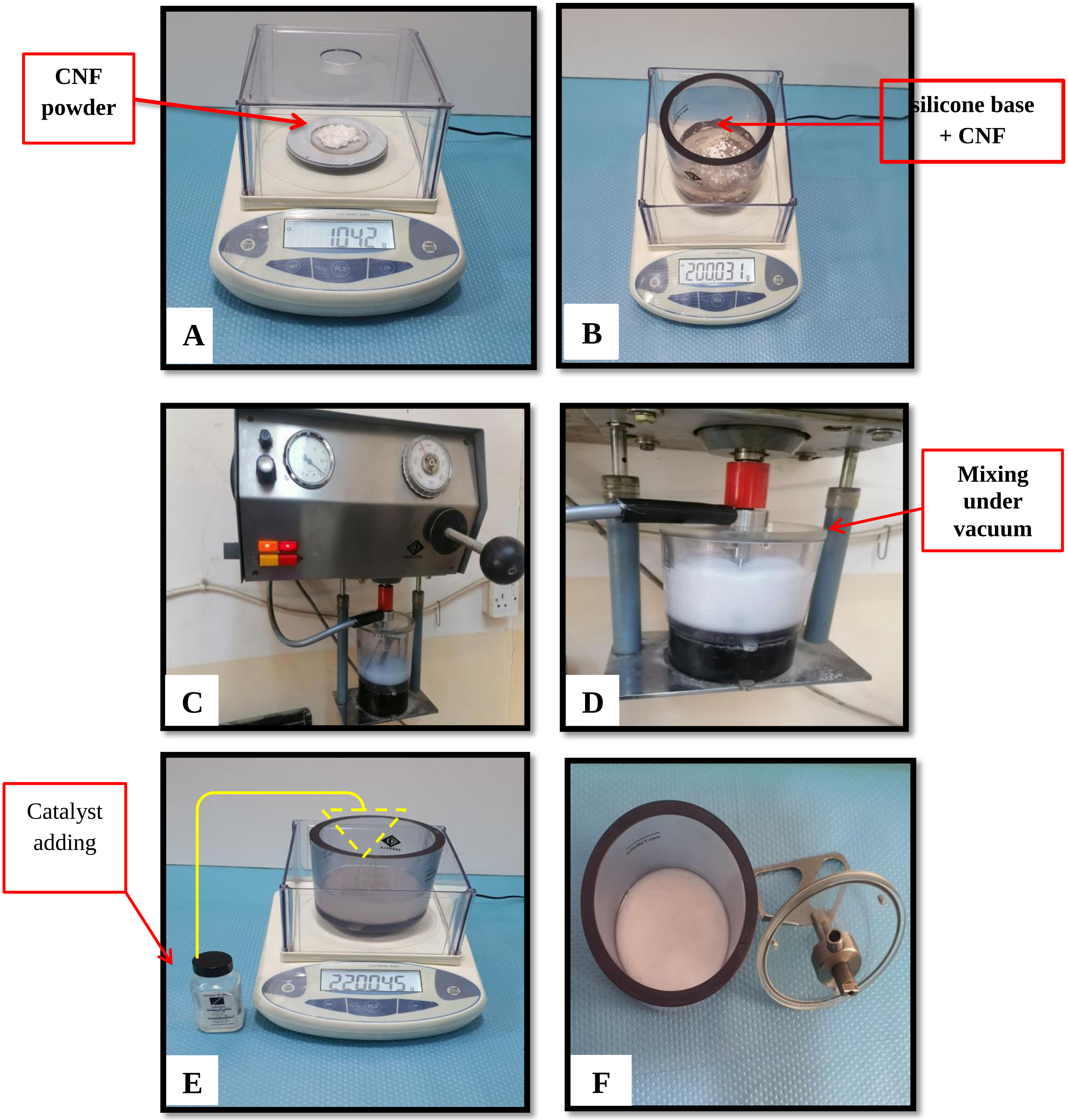

Mixing procedures: A, The Nano cellulose fibers measured in the electronic scale. B, silicone base and CNF addition measured in the electronic scale. C, The silicone base mixed with the CNF powder D, modified base mixing with(-10) bar vacuum. E, the catalyst (part B) adding to the mixture according to the weight. F, Homogenous mixture free air bubble ready for pouring.

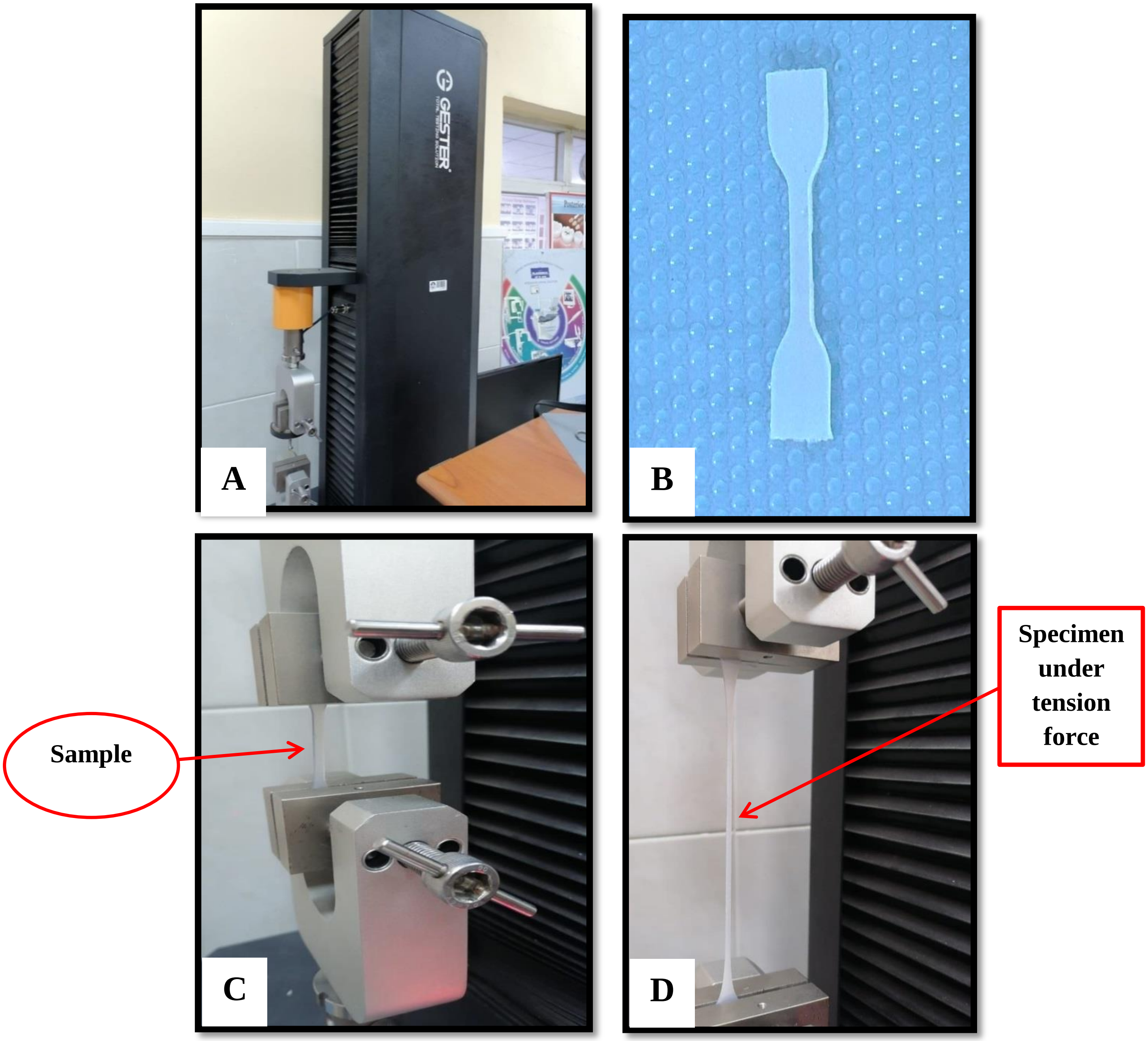

Tensile strength test, A: Gester testing machine B: shape of specimens used for tensile strength test. C:specimen hold by device D:specimen under tension force.
